# Delirium after Deep Brain Stimulation in Parkinson's Disease

**DOI:** 10.1155/2021/8885386

**Published:** 2021-02-01

**Authors:** Hanyi Li, Shunchang Han, Juan Feng

**Affiliations:** Department of Neurology, Shengjing Hospital of China Medical University, No. 36 Sanhao Street, Shen Yang 110004, China

## Abstract

Deep brain stimulation is a primary treatment method that improves motor and motor complications in patients with advanced Parkinson's disease. Delirium is a common and serious complication following deep brain stimulation. However, the clinical attention toward this complication remains insufficient. Advanced age, cognitive decline, and the severity of the disease may all be risk factors for delirium. The presence of delirium may also affect cognitive function and disease prognosis. Neurotransmitters such as acetylcholine and dopamine may be involved in the occurrence of delirium. Furthermore, inflammation, the effects of microlesioning of local nuclei, and brain atrophy may also play roles in the onset of delirium. Nonpharmacological therapy appears to be the primary treatment for postoperative delirium in Parkinson's disease. The current article reviews the pathogenesis, epidemiology, prognosis, and treatment of delirium following deep brain stimulation in Parkinson's disease to help clinicians better understand this common complication and to prevent, identify, and treat it as soon as possible, as well as to provide more accurate treatment for patients.

## 1. Introduction

Parkinson's disease (PD) is one of the most common degenerative diseases of the nervous system. More than six million people worldwide suffer from Parkinson's disease [1]. The primary clinical symptoms of PD are slow movement, rest tremor, muscular rigidity, and abnormal postural gait. Currently, the primary treatment for PD is dopamine replacement therapy, which has a significant effect on the motor symptoms of PD. However, as the disease progresses, PD frequently presents with uncontrollable motor complications such as motor fluctuations and dyskinesia [1]. Deep brain stimulation (DBS) is a well-known treatment that can help to manage motor fluctuations and dyskinesia better than medical therapy only [2]. Deep brain stimulation (DBS) involves the implantation of stimulation electrodes to a specific brain region through stereotactic surgery in order to achieve therapeutic effects by generating high-frequency electrical stimulation for that particular brain region. The surgical side effects such as intracerebral hemorrhagic and infections are rare, with incidence rates ranging from 0.2% to 5% [3]. However, excluding any immediate complications of the operation, postoperative neuropsychiatric complications, including suicide, postoperative depression, postoperative euphoria, and/or hypomania, are common after DBS surgery [4].

Postoperative delirium is one of the most common neuropsychiatric complications following DBS surgery, with incidence rates reaching 42.6% [5]. Previous research has indicated that delirium is associated with the deterioration of cognitive functions, worsening of motor symptoms, and a more poor prognosis [6, 7]. However, until now, this complication has not been fully evaluated; indeed, only a few studies focus on this complication. Identifying the causal risk factors in patients with delirium following DBS, early detection, and treatment of this complication is essential for an improved outcome of patients after DBS. In the current review, the epidemiology, risk factors, and pathophysiology and treatment of delirium after deep brain stimulation are examined.

## 2. Definition and Clinical Criteria

Delirium is characterized using either the Diagnostic and Statistical Manual of Mental Disorders, Fifth Edition (DSM-5) [8] or the 10th revision of the International Statistical Classification of Diseases and Related Health Problems (ICD-10) [9]. Delirium is characterized by an acute disturbance in attention and awareness that fluctuates, accompanied by an additional disturbance cognition per the DSM-5. Delirium usually develops within 72 hours after surgery taking, in some populations, up to five days. Delirium can present as hypoactive, as hyperactive, or as mixed forms. At present, the recognition of hypoactive delirium is still insufficient. The hypoactive delirium generally has a poor prognosis, which is plausibly related to the failure of the clinician to make a timely diagnosis, leading to the delay of diagnosis and treatment [10]. The current gold standard for the diagnosis of delirium is for a professional to use the DSM-V or ICD-10 diagnostic criteria. However, both of these diagnostic criteria are complicated and it is difficult to screen patients quickly in clinical application. Currently, two highly sensitive screening tools are recommended; the Nursing Delirium Screening Scale (Nu—DESC) [11] and the Confusion Assessment Method (CAM) [12, 13].

Various definitions of delirium after DBS in Parkinson's disease have been studied. Some studies use the Confusion Assessment Method-Intensive Care Unit (CAM-ICU) to identify delirium. This method includes four characteristics: (1) acute onset and fluctuating condition; (2) inattention; (3) disordered thinking; (4) a change in consciousness. If the patients exhibit the first two combined with either disordered thinking or a change in consciousness, they are to be categorized to have delirium [14, 15]. Several other studies have not used definitive scales. One study defined delirium as varying degrees of temporal disorientation, spatial disorientation, and cognitive impairment beginning an hour after surgery, with a shorter duration of resolution [16]. Another study described delirium as any degree of hallucination, confusion, or spatial disorientation [17]. In addition, a case report described a patient who, under delirium after DBS surgery, showed delusions, hallucinations, confusion, and very aggressive behavior within 48 hours following the implantation and activation of the electrode [18].

These studies demonstrated that the common symptoms of delirium after DBS are hallucinations, delusions, confusion, and disorientation. However, these symptoms overlap with the mental symptoms of PD patients, which makes it difficult to identify delirium in these patients. Therefore, the acute occurrence of confusion and disorientation is generally more helpful for the identification of PD delirium than the fluctuating hallucinations. Available scores have not currently been validated in patients with PD. In the future, the sensitivity of the existing scale to the identification of delirium in PD patients needs to be further analyzed. The development of a scale more suitable for Parkinson's disease is also prudent.

## 3. Prevalence

Currently, studies on postoperative delirium (POD) following DBS are scarce (Table 1). Furthermore, the results from published studies are inconsistent. For instance, Carlson et al. utilized 59 patients undergoing DBS surgery for the subthalamic nucleus (STN) to demonstrate that 22% of DBS patients had delirium after electrode implantation and 10% had delirium after their second operation [17]. Similar data were reported in Wang et al., who designed a retrospective study that included 165 patients undergoing bilateral subthalamic nucleus surgery, using the CAM-ICU to diagnose delirium and reported an incidence of postoperative delirium after DBS surgery of 19.4% [14]. Other studies, however, have come to quite different conclusions. Abboud et al. [19], for example, used the DSM-5 diagnostic criteria and reported a relatively low incidence in a study of 130 patients, with only seven cases (5.8%) showing postoperative delirium. It is important to note that the DSM-5 diagnostic criteria are a formal diagnostic method for delirium. Tanaka et al. [5], however, reported the incidence of postoperative delirium as high as 42.6% out of 61 patients included in the study, among whom 52 patients were targeted in the subthalamic nucleus (STN)-DBS, eight at the globus pallidus interna (GPi)-DBS, and others at the ventralis intermedius (VIM)-DBS. The study did not use reliable diagnostic tools; rather, they defined POD as an event involving hallucinations and delusions, among other criteria, that occurs within 14 days after surgery.

There are several possible reasons that explain differences in the research. First, different studies utilized different definitions of delirium. Some studies used standardized scales or the DSM-IV diagnostic criteria for delirium identification, while others did not standardize the diagnosis of delirium in patients, potentially overestimating the incidence of delirium. Second, different sample sizes in different studies may lead to large differences in results. Third, different countries and hospitals may require different inclusion and exclusion criteria for patients undergoing DBS surgery, resulting in different results. In 2006, scholars conducted a meta-analysis of the results of PD treatment with STN-DBS in 778 patients, reporting an incidence of postoperative delirium of 15.6% [3]. In addition, the incidence of delirium following DBS surgery at different areas of the brain has not been studied. Future studies should utilize a larger sample size and a more standard assessment of delirium to accurately study the probability of delirium (Tables 1 and 2).

## 4. Risk Factors

### 4.1. Age

Previous studies have shown age to be a major risk factor for postoperative delirium (Table 2) [24–27].

Similar results were found in patients following DBS surgery. Paim et al. [16] studied 49 Brazilian patients who received bilateral STN electrode implantation, focusing only on postoperative confusion, but did not use a scale for a definition of postoperative confusion. They reported that those who with postoperative confusion were older (63.2 ± 7.8 years vs. 55.4 ± 9.1 years, *p*=0.009) and had a longer course (16.5 ± 5.1 years vs. 13.2 ± 4.2 years, *p*=0.027) of the disease. Two other studies from China reported similar results. A study of 229 PD patients over the age of 50 who underwent DBS surgery [15] used the CAM-ICU to evaluate delirium and showed that older patients are more prone to delirium (62.01 ± 6.30 vs. 65.43 ± 6.57, *p*=0.002) and that age is an independent risk factor of POD (OR = 1.074, 95% CI: 1.012, 1.140). The same team reported similar results in another article with a total of 165 PD patients who received bilateral STN-DBS [14]. Patients in that study with postoperative delirium were slightly older (62.78 ± 9.30 vs. 60.06 ± 9.11), but not statistically significant so (*p*=0.133).

### 4.2. Disease Severity and Nonmotor Symptoms

Abboud et al. [19] utilized 130 American patients with PD who had received STN-DBS surgery, including implanted unilateral and bilateral electrodes. The UPDRS III for postoperative delirium patients averaged 37.8 ± 2.04 points, while delirium-free patients averaged 19.7 ± 7.5 points. This study also demonstrated that, for each one-unit increase in the UPDRS III or MDS-UPDRS III score at the opening stage, the probability of postoperative delirium increased by 10%. Furthermore, this study also showed that postoperative delirium was more common in patients with preoperative falls and balance disorders (10.2% vs. 1.6%).

Patients with PD often have a variety of nonmotor symptoms. Some nonmotor symptoms, such as dementia and hallucinations, are a sign of progressive Parkinson's disease. Studies have shown that nonmotor symptoms are also a risk factor for postoperative delirium. Carlson et al. [17] demonstrated that preoperative hallucinations are closely related to postoperative delirium. Wang et al. [14] reported that a poorer cognitive status, higher quantity of motor symptoms, and poorer sleep quality were all related to a greater risk of POD. The same team conducted another study utilizing Parkinson's patients over the age of 50 and reported similar results [15]. Additionally, in a study of 21 patients receiving STN-DBS, Radziunas et al. [22] showed that, although there was no significant difference in global cognitive function between delirium and nondelirium patients, delirium patients had a poorer executive function in trail making, drawing, digit symbol coding, and symbol copying. This suggests that the frontal lobe and other brain regions that are closely related to executive function and may be involved in the occurrence and development of delirium. Another study that involved 96 patients undergoing bilateral STN-DBS surgery [21] similarly concluded that patients with postoperative confusion had poorer frontal lobe function. The same study also found a strong link between depression and postoperative confusion.

### 4.3. Brain Atrophy

Studies have shown that brain atrophy also plays an important role in postoperative delirium. Radziunas et al. [22] studied the brain structure of 22 patients following DBS using Voxel-based brain MRI morphometry (VBM) techniques and showed that, compared with the patients without postoperative neuropsychiatric complications, the thickness of the bilateral common cortex was significantly smaller, including the bilateral frontal lobe (tail of the middle frontal gyrus and anterior central gyrus), temporal lobe (inferior temporal gyrus and middle temporal gyrus), and parietal lobe (posterior central gyrus, parietal gyrus, and superior marginal gyrus). Furthermore, there was no significant difference in overall white matter thickness between the two groups. However, the thickness of white matter in patients with postoperative psychiatric symptoms was, indeed, smaller in the left tail middle frontal gyrus, left tongue gyrus, left parathyroid gyrus, left precuneus, and right parathyroid gyrus. Tanaka et al. [5] also showed that white matter volume was negatively correlated with POD duration, particularly the temporal stem white matter atrophy, which was significantly correlated with POD duration.

### 4.4. Surgical Effects

Some surgical factors can also contribute to delirium. Gologorsky et al. [23] conducted an analysis of 81 patients following DBS and showed that intraventricular wall invasion during surgery is a risk factor for postoperative delirium. Another study showed that patients with postoperative delirium had a higher incidence of postoperative cerebral hemorrhage than those without [16]. In addition, Wang et al. found a relationship between postoperative brain edema and postoperative delirium [14]. In addition, several studies have suggested that surgical procedures do not seem to have a clear effect on postoperative delirium. Abboud et al. [19] showed that neither the operative side nor the number of electrodes passes during the operation were associated with delirium. In addition, the duration of operation was found to be independent of the occurrence of delirium. This suggests that surgical factors do not seem to play a significant role in the onset of delirium. However, some surgical complications, such as bleeding and edema, may contribute to delirium, which should be taken seriously. Therefore, it is important to avoid the invasion of the lateral ventricle wall during electrode implantation.

Preoperative assessment of relevant risk factors such as old age, hallucinations, cognitive function, brain atrophy, etc., in patients with DBS can help to identify high-risk patients with postoperative delirium in the early stage, develop more appropriate surgical and anesthetic plans, and identify and deal with related issues in the early stage after the operation. In addition, the ESA (European Society of Anaesthesiology) [28] guidelines outline POD associated risk factors, such as the use of choline drug resistance, alcohol causes of cognitive impairment, length of surgery and postoperative pain, system function impairment and weakness, malnutrition, hypoalbuminemia, among others. In spite of these, research on patients undergoing DBS surgery is still lacking, but these risk factors should be carefully considered by the clinician.

## 5. The Influence of POD on Disease Prognosis after DBS

Postoperative delirium typically resolves spontaneously after a few days. Radziunas et al. reported the duration of delirium following DBS ranged from only one to four days [22]. According to Masataka et al. [5], the average onset of POD is 1.57 days. It is important to note that delirium rarely turns into a serious and persistent mental disorder. However, postoperative delirium still has an impact on the clinical prognosis of DBS patients.

### 5.1. Extended Hospital Stay

Previous studies of hip and vascular surgery patients have shown that postoperative delirium and postoperative confusion are associated with longer hospital stays and higher costs [29, 30]. Similar results were found in Aboud et al.'s study [19] on patients with PD following DBS, which demonstrated that 15.8% of patients had a hospital stay longer than two days and that hospitalization time in all patients with postoperative disorders was prolonged. A similar study by Carlson et al. [17] showed that postoperative delirium was an important cause of prolonged hospitalization in PD patients who received DBS surgery.

### 5.2. Cognitive Function Affect and Long-term Prognosis

Previous studies have shown that for PD patients, the occurrence of delirium increases the risk of dementia, dyskinesia, and death [31] and also increases the risk of disease progression [7]. In addition, studies of patients with hip, heart, or colorectal surgery have shown that POD is independently associated with worse clinical outcomes, spur cost, and increased mortality [32–35]. A recent meta-analysis also suggests that delirium is significantly associated with long-term cognitive decline [36]. Moreover, there is growing evidence that the duration of delirium, not just its occurrence, plays a crucial role in a variety of adverse, long-term outcomes, including cognitive impairment and death [37–39]. One study of patients also found that following DBS, the mRS score of patients with POD was significantly higher at two years after DBS than that of patients without POD (3.69 ± 1.19 vs. 2.94 ± 0.95, *p*=0.0087). In addition, the POD duration after DBS was significantly correlated with mRS scores [5]. Pilitsis et al. [21] reported that postoperative confusion tends to exacerbate the cognitive decline. The authors followed 96 patients with bilateral STN-DBS surgery for an average of five months and showed that patients with postoperative confusion performed worse on the Mattis DRS Total Score, Story Memory test, and Immediate Recall.

Currently, there is still a lack of relevant studies on the long-term postoperative cognitive impairment and mortality change of patients with DBS associated with POD, and relevant research efforts may need to be strengthened in the future to more comprehensively analyze and understand the impact of the POD on the prognosis of DBS.

## 6. Occurrence Mechanism of POD following DBS

Delirium is a complex clinical manifestation associated with multiple risk factors and its pathogenesis is still unclear. A large number of studies have proposed a neurotransmitter hypothesis and/or a neuroinflammatory hypothesis, but a single hypothesis may not be able to independently explain the pathogenesis of delirium [40]. Additionally, studies have shown that delirium may be closely related to neural network abnormalities and genetic susceptibility. The delirium induced by DBS surgery may have a unique pathophysiological mechanism, which may be closely related to the brain areas directly damaged by surgery and brain atrophy during surgery.

### 6.1. Neurotransmitter Hypothesis

Neurotransmitter imbalance is considered to be the major pathogenesis of delirium. The occurrence of delirium may be related to various neurotransmitters, such as acetylcholine, dopamine, gamma-aminobutyric acid, melatonin, tryptophan or serotonin, glutamate, epinephrine, or norepinephrine, along with many others [40]. However, the neurotransmitters most closely related to delirium are acetylcholine and dopamine. The cholinergic system, regulated by an open circuit connected to the basal ganglia, affects a wide range of functions including cognition, attention, gait, and postural stability. Research has shown that increased serum anticholinergic activity is independently associated with delirium [41]. Similarly, the use of anticholinergic drugs is closely related to the occurrence of delirium [42, 43]. The 2017 guidelines for postoperative delirium also suggest that the preoperative use of anticholinergic drugs is an independent risk factor for postoperative delirium. Dopaminergic abnormalities are also closely related to delirium and a case report describing anticholinergic agent induced delirium is similar to that reported in the case of levodopa administration [43]. In addition, dopamine system genes such as the transporter gene and the dopamine receptor 2 gene are also closely associated with the occurrence of delirium [44]. Parkinson's disease may have a variety of neurotransmitter abnormalities, such as dopamine, acetylcholine, and 5-hydroxytryptophane, among others. Particularly in the advanced stages of Parkinson's disease, the above neurotransmitter abnormalities may be more complex, resulting in advanced Parkinson's disease with increased brain vulnerability. Currently, DBS surgery is frequently used in patients with advanced PD. In this group of patients, complex neurotransmitter transduction and fragile brain function may be the primary pathogenesis of postoperative delirium following DBS.

### 6.2. Neuroinflammatory Hypothesis

The neuroinflammatory hypothesis is another hypothesis that has received significant attention, particularly in recent years. Neuroinflammation is not only closely related to a variety of neuroimmune-related diseases, but it was also recently found that dementia, PD, and other neurodegenerative diseases are inextricably related to neuroinflammation. Neuroinflammation may also play a crucial role in the progression of delirium. A study of 94 patients demonstrated that C-reactive protein levels predicted the onset and recovery of delirium [45]. Other inflammatory factors, such as IL-6 and IL-8, are also associated with delirium. In a study involving 156 patients [46], IL-6 was strongly associated with the duration of delirium in nondementia patients. Similarly, in another study involving 144 patients with ischemic stroke [47], an increase in plasma IL-6 was strongly associated with the occurrence of poststroke delirium. In addition, the results of the autopsy in nine delirium patients and six in the control group showed that the astrocyte activity and IL-6 immune activity were higher in delirium patients [48]. Inflammatory cytokine disorders can lead to nerve damage through a variety of mechanisms [49], including neurotransmitter changes, cell apoptosis, and the abnormal activation of microglia and astrocytes. Currently, there are no existing studies focusing on the relationship between inflammatory factors and postoperative delirium following DBS. However, inflammation plays an important role in the pathogenesis of delirium and PD, so it is reasonable to believe that inflammation also plays an important role in the occurrence and development of delirium following DBS.

### 6.3. Direct Damage of Electrode to Subthalamic Nucleus

Delirium following DBS may have a unique mechanism that is different from another postoperative delirium. Existing studies of patients who have had DBS surgery have shown that delirium after DBS electrode implantation is much higher than that after the second surgery [17]. This suggests that, compared with other nonintracranial operations, the surgical implantation of electrodes may have a direct psychiatric effect on the microdamage of STN. Several cognition-related and emotion-related circuits are in the basal ganglia multiple circuits [50], including the dorsolateral prefrontal circuit (DPC), lateral orbitofrontal circuit (LOC), and basal hominins ganglia thalamocortical limbic circuit. These circuits are closely related to cognitive and other high-level processes, motion, motivational, and affective processes. Any abnormality within these pathways may lead to cognitive changes and mental symptoms. The STN has, both anatomically and functionally, a central position within these circuits, so any damage to this area can impair emotions and emotional control. In addition, studies have shown that STN plays a pausing role in the neural loop [51]. This pause function helps the brain forget unnecessary information and better store and encode new information. It is plausible that a loss of the suspend function causes changes in the neuronal activity of the prefrontal cortex through the hyperdirect pathway. Therefore, direct damage to STN from DBS electrodes may trigger neuronal signal dyssynchrony in the corticothalamic neural network. An abnormal nerve conduction signal of this neural network may be related to delirium following DBS.

### 6.4. Functions of Brain Regions

Certain brain regions may be involved in the pathogenesis of postoperative delirium following DBS. Gray matter thickness, white matter volume, and brain surface area in the caudal middle frontal area of the left hemisphere were all reduced in patients with post-DBS delirium, suggesting that this area may be involved in the mechanism of post-DBS delirium [22]. Furthermore, previous studies have shown that theta band activities in the middle frontal cortex are an important part of the cognitive control system [52]. Another single-photon emission computerized tomography (SPECT) study demonstrated that the medial prefrontal lobe is dominant in the frontal-striatum-thalamus circuit and is responsible for cognitive and executive functions [53] and is also strongly associated with mood. This mechanism may contribute to the development of delirium. In addition, temporal lobe atrophy is closely related to the duration of POD [5], suggesting that the temporal lobe, a crucial part of the limbic system, may also play a role in the onset of post-DBS delirium.

At present, the related mechanism of delirium after DBS is still unclear and needs to be further studied.

## 7. Prevention and Treatment of Delirium after DBS

### 7.1. Prevention of Postoperative Delirium

Factors that predict the progression of delirium include existing hallucinations, advanced age, and a longer course of illness. Family members should be advised of the increased risk of delirium and a treatment plan should be developed in advance for patients with higher risk factors. However, despite the high incidence of post-DBS delirium, electrode implantation in high-risk patients should not be discontinued, as the benefits outweigh the risks [22]. In addition, during the operation, particularly for elderly PD patients with temporal stem atrophy, electrode tracks involving the caudate head or lateral ventricle wall should be avoided [5, 23]. Shortening the duration of surgery and electrode implantation under general anesthesia may also help prevent delirium following DBS, in spite of the lack of evidence for this.

### 7.2. Nonpharmacological Management

A primary aspect of delirium management is early detection. Current research shows that the rate of missed diagnosis in postoperative delirium patients is high, up to 60% [54]. The guidelines suggest that doctors and nurses should use simple screening tools such as the CAM and NU-DESC to diagnose patients early so that they can be treated early [28]. The preferred treatment of delirium is nondrug therapy and returning the patient to a familiar home environment, particularly in mild delirium. Family medication regimens are usually gently readjusted by caregivers and close family; caregiver involvement typically works well. For benign hallucinations or disturbances of consciousness, such as sunset phenomena (night delirium and mild delirium), observation is the best treatment. Physical restraint should only be considered if the patient's mental symptoms affect the safety of themself or others [17]. A recent review summarizes the management of delirium in PD [55], including frequent visits by family members and nursing staff, getting bright light during the day and darker light at night, as well as avoiding noise, reestablishing circadian rhythms to improve sleep, avoiding inhalation and maintaining nutrients, and preventing falling. In addition, previous studies on delirium in PD patients have shown that several of the drugs given to PD patients are associated with an increased risk of delirium and the guidelines suggest that drug review should be part of delirium management. Dosage reduction or discontinuation of high-risk medications that may cause delirium is appropriate [56].

### 7.3. Pharmacological Management

Previous guidelines for POD recommended the use of haloperidol or atypical antipsychotics. However, in PD patients, haloperidol was associated with a significant increase in extrapyramidal symptoms compared with other atypical antipsychotics [57]. Haloperidol is currently banned because of the risk of extrapyramidal side effects and the malignant syndrome of antipsychotics, as suggested in the MDS guidelines [56] for psychiatric symptoms of PD. These guidelines also evaluated three atypical antipsychotics. Clozapine is considered effective against hallucinations in PD. In 2007, a meta-analysis suggested that clozapine may be the only drug with demonstrated efficacy in treating Parkinson's psychiatric symptoms [58], but is problematic in routine clinical applications because side effects require specialized monitoring. Quetiapine is considered to be poorly documented, but since it does not require any specialized monitoring, it is a viable option, while olanzapine carries an unacceptable risk of motor function degradation. Currently, some researchers believe that quetiapine is the safest choice for delirium treatment in PD patients [59]. In patients who received DBS, quetiapine has been shown to be able to control delirium following DBS surgery [18, 22]. In addition, a case report showed that Zolpidem improved psychiatric symptoms after DBS. Therefore, it appears to be an attractive drug for post-DBS delirium; however, further research is needed [60].

## 8. Conclusion

DBS is a good treatment for patients with Parkinson's disease in the middle and advanced stages. Previous studies have demonstrated that this treatment regimen can notably improve motor symptoms, improve multiple motor complications that cannot otherwise be controlled by drugs, and is effective for some nonmotor symptoms. The side effects of DBS surgery are few and intracranial hemorrhage is more common, but the overall risk is lower. However, previous studies and clinical practice have shown that delirium frequently occurs following DBS surgery. Although the duration of delirium is typically short with a quick recovery, delirium may influence the long-term motor symptoms, cognitive status, and adverse outcomes of patients following DBS surgery, suggesting a need for close attention to be paid by clinicians. In addition to basic screening for patients before DBS and to screening for DBS-related contraindications, a full understanding of the risk factors, such as mild cognitive impairment, minor hallucinations (particularly minor hallucinations such as illusions and presence perception), as well as the patient's advanced age, disease duration, and brain atrophy is also required. For patients with a high risk of delirium (such as patients with advanced age, hallucinations, cognitive dysfunction, or severe brain atrophy), the operation plan should be made flexible (care needs to be taken to avoid the invasion of the lateral ventricle wall during electrode implantation; in addition, shortening the duration of anesthesia may also be a good method). At the same time, screening for postoperative delirium in high-risk patients should be more frequent. In addition, patients should restart the use of anti-Parkinson drugs as soon as possible after the operation so as not to delay the drug and aggravate the occurrence of delirium. In the future, more attention should be paid to the mental disorders of PD patients, particularly the short-term delirium following DBS and the long-term symptoms of mental disorders. More research is needed to be able to provide more personalized and appropriate treatment for Parkinson's disease and improve the quality of life of patients with advanced PD.

## Figures and Tables

**Table 1:** Recent studies on postoperative delirium or postoperative confusion following DBS.

Year	Country	Number of cases	Target	Assessment of delirium	Incidence rate (%)	Primary research results
2003 [20]	France	49	STN	Unsystematic evaluation	24.4	The motor function of PD patients receiving STN-DBS improved significantly at 5 years
2005 [21]	America	96	STN	Unsystematic evaluation	9	Depression and prefrontal dysfunction are closely related to DBS
2014 [17]	America	59	STN	Unsystematic evaluation	22	Advanced age, preoperative hallucinations are associated with POD
2020 (2018) [22]	Lithuania	22	STN	4AT scale	18.2	Patients with POD had smaller bilateral cortex thickness and worse nonverbal executive function
2018 [5]	Japanese	61	STN: 52;	Unsystematic evaluation	42.6%	Age and total white matter volume were related to POD duration
			GPi: 8;			
			VIM: 1			
2019 [16]	Brazil	49	STN	Unsystematic evaluation	26.5	POD patients were older, had a longer course of the disease, and had a larger width of the third ventricle.
2019 [14]	China	165	STN	CAM-ICU	19.4	The higher the age and the worse the cognitive status, the higher the probability of POD occurrence.
2020 [19]	America	130	STN: 121	DSM-IV	5.8%	Preoperative tremor and UPDRSIII score, as well as falls and balance disturbances, are associated with postoperative delirium
			GPI: 7			
			VIM: 2			
2020 [15]	China	229	STN	CAM-ICU	20.52	Gender, age, PDSS, and preoperative cerebral ischemia have certain effects on the occurrence and development of POD

CAM-ICU: Confusion Assessment Method-Intensive Care Unit; 4-AT: Assessment Test for Delirium and Cognitive Impairment Scale; DSM-IV: Delirium is characterized using either the Diagnostic and Statistical Manual of Mental Disorders, Fourth Edition.

**Table 2:** Risk factors associated with delirium following DBS.

Classification	Risk factor	Reference
	Age	Paim et al.2019 [16]
		Tanaka et al.2018 [5]
		Carlson et al. 2013 [17]
	Severity of disease	Abboud et al. 2020 [19]
		Wang et al.2019 [14]
	Disease duration	Paim et al.2019 [16]
		Carlson2013 [17]
	Comorbidity	Paim et al.2019 [16]
Motor symptoms	Absence of tremors	Abboud et al. 2020 [19]
	Falls/balance dysfunction	Abboud et al. 2020 [19]
Nonmotor symptoms	Cognitive decline	Pilitsis et al. 2005 [21]
		Radziunas, et al. 2020 (epub2018) [22]
		Wang et al.2019 [14]
	History of hallucinations	Carlson et al.2013 [17]
	Sleep disorders	Wang et al.2019 [14]
Imaging abnormality	Greater third ventricle width	Paim et al.2019 [16]
	Brain atrophy	Radziunas, et al. 2020 (epub2018) [22]
		Tanaka et al.2018 [5]
		Wang et al.2019 [14]
Surgical effect	Ventricular wall transgression	Gologorsky et al. 2011 [23]
	Postoperative brain edema	Wang et al.2019 [14]

## Data Availability

No data were used in the study.
